# MMLKG: Knowledge Graph for Mathematical Definitions, Statements and Proofs

**DOI:** 10.1038/s41597-023-02681-3

**Published:** 2023-11-10

**Authors:** Dominik Tomaszuk, Łukasz Szeremeta, Artur Korniłowicz

**Affiliations:** University of Bialystok, Faculty of Computer Science, Bialystok, 15-245 Poland

**Keywords:** Computer science, Scientific data

## Abstract

Nowadays, Knowledge Graphs (KGs) are important and developing in different areas. However, there is a lack of genuinely interoperable datasets representing mathematics that allow for information exchange between datasets in the Web ecosystem. In this paper, we address this matter based on the Mizar Mathematical Library (MML), a collection of articles written in the Mizar language. MML includes definitions and theorems with proofs to which authors can easily refer from newly written Mizar articles. However, extracting information directly from Mizar scripts by external projects is not very straightforward. Therefore, we propose a new data storage and retrieval approach based on the Knowledge Organization System (KOS) model and the KG concept that provides a way to organize and access knowledge. We present *Mizar Mathematical Library Knowledge Graph* (MMLKG), a thesaurus for describing mathematical objects. MMLKG supports semantic interoperability and allows linking data from different sources, e.g., Wikidata. Moreover, it satisfies the FAIR data principles. The data is publicly available via a Cypher endpoint.

## Background & Summary

The Mizar Mathematical Library (MML)^1^ is a repository of mathematical papers written in the Mizar language and computer-verified by the Mizar proof-checker^2^. The Mizar language is designed to be readable for mathematicians and processable by computers. It is *a closed language* in the sense that it consists of a predefined set of reserved words used for writing papers, but it is *an open language* in the sense that users can introduce new mathematical concepts and prove theorems about them.

MML was established in 1989 and has been growing regularly every year. In 2023, it contains over 1,400 articles with over 13,000 definitions and over 65,000 theorems within various mathematics and computer science branches, including recent formalizations on set theory^3^, algebra^4^, Hilbert spaces^5^, fuzzy sets^6^, number theory^7^, and many others. The still bigger size of the library makes the searching information through the library more and more time-consuming. In consequence, dedicated tools, like MML Query^8^, or solutions based on the Latent Semantic Indexing^9^ for presenting and indexing the library’s content and searching for required information become indispensable.

For many years the content of MML has been a subject to representing mathematical knowledge in various formats, e.g.: as papers published in the Formalized Mathematics journal (https://sciendo.com/journal/FORMA), as hypertext documents (https://mizar.uwb.edu.pl/version/current/html/articles.php), in the OMDoc format^10,11^ designed for representing Science, Technology, Engineering, and Mathematics (STEM) documents and in formats used by automated theorem provers^12,13^. To facilitate the translation of Mizar documents stored in MML to external formats, a sequence of XML-based languages representing various information accessible at different stages of processing given articles have been developed. The most representative languages are *Weakly Strict Mizar*^14,15^, which allows representing the syntactic tree of the analyzed article; *More Strict Mizar* which extends the former with information about the sentence and variable identifiers; and *Even more Strict Mizar*^16^ (ESX), which allows an exact description of the syntactic structure of an article to be represented in connection with the resolved semantic information about all mathematical concepts used in Mizar articles.

The *Even more Strict Mizar* format is the basis for building our proposal, the Mizar Mathematical Library Knowledge Graph (MMLKG). Our proposal leverages FAIR principles^17^ to create a graph dataset that allows users to seamlessly query and reason over some of the most prominent interrelated definitions and proven theorems. We follow the Linked Data principles^18^. Moreover, MMLKG is built as a thesaurus on the CoVoMe methodology^19^.

We aim to provide enough details to enable researchers, application developers, mathematicians, and others interested in utilizing the Mizar data in their work. The usefulness of our dataset lies in the fact that it includes classification and relationships between mathematical entities. All concepts, collections, and terms contain semantic relationships, so all mathematic entries are available to semantic reasoning tools that harness the classification hierarchy. The dataset and the endpoint can facilitate further research on searches to be expanded and redefined, present references to resources with content related to those directly retrieved in the search, or suggest new search terms. An additional value of the MMLKG dataset is that it is shareable, extensible, and easily reusable. Resources can be described in collaboration with other datasets and linked to data contributed by other communities. Moreover, we provide a web endpoint that allows us to query and visualize our data. Leveraging knowledge graphs enhances semantic search in mathematics, improving user comprehension for discovering scientific materials, aiding query expansion, and supporting new mathematical works. Additionally, our knowledge graph benefits education, database management, and scientific research by organizing resources, integrating data, and creating concept maps to enrich mathematical knowledge and enhance research quality.

We proceed as follows: first, we describe our data collection and present the schema. Next, we discuss the quality of our data. Then, we outline usage notes for researchers. We plan to update the dataset quarterly. Researchers and developers can also use our pipeline to reconstruct the dataset, which requires several hours to process the data sources.

## Methods

### KOS and CoVoMe methodology

The term Knowledge Organization System (KOS), intended to encompass all schemes for organizing information and managing knowledge^20^, perfectly fits the Mizar data. According to CoVoMe^19^, we define MMLKG using six steps:

1. Determine the type of controlled vocabulary: In this step, we specify what type of KOS they are building. We choose major types of controlled vocabulary which are based on features such as structure, complexity, relationships among terms, and historical function. Then we choose the concrete type of controlled vocabulary.
2. Define the concepts: In this step, we denominate ideas, meanings, or objects and events. We choose a strategy for identifying the concepts: bottom-up, top-down, or middle-out. Then, we define the concepts according to the previously selected strategy.
3. Define the labels and notations: In this step, we define the labels and notation used in our controlled vocabulary. This includes specifying synonyms that may be used. Then, we define any notation – codes used to represent the concepts. The goal of this step is to ensure that the controlled vocabulary is clear and unambiguous, and that all terms are consistently defined and used throughout the vocabulary.
4. Define the semantic relations: In this step, we define the semantic relationships between the concepts in our controlled vocabulary. We define the hierarchical relationships between concepts and specify associative relationships between concepts, such as related terms. The goal of this step is to ensure that the relationships between concepts are clearly defined and accurately reflect the relationships between the concepts in the real world.
5. Define groups of concepts: In this step, we define groups of concepts that are useful, where collected concepts have something in common and it is convenient to group them. The collections can be nested.
6. Integrate with other sources: In this step, we integrate our controlled vocabulary with other sources to ensure consistency and interoperability.

According to CoVoMe, we chose a thesaurus as a controlled vocabulary for MMLKG. We chose a middle-out strategy. Based on the Mizar collection stored in ESX documents, we define concepts and their labels. We describe notations as the means of access to the concepts they contain. ESX data contains concepts represented by terms organized so that the relationships between concepts are made explicit. These relationships can be hierarchical (and asymmetric) or associated (and symmetric). Moreover, it is possible to group different concepts into groups. We can also provide links to other sources to integrate them.

### FAIR Principles

The FAIR principles^17^ are gaining increasing traction across all scientific domains. We describe the FAIR principles, slightly rephrasing the original wording in some cases for brevity:

F. *Findability* refers to the ease with which external tools that might benefit from the knowledge graph can initially locate it.

A. *Accessibility* refers to the ease with which external tools can access knowledge.

I. *Interoperability* refers to the ease with which the knowledge graph can be exploited in conjunction with other data sources using standard tools.

R. *Reusability* refers to the ease with which the data from the knowledge graph can be reused in conjunction with other data sources.

To ensure *findability*, we assign a globally unique and persistent identifier (e.g., UUID) for data and metadata. Furthermore, machine-generated metadata is attached to MMLKG (e.g., the number of definitions and the creation date). Finally, the search interface allows users to search whole graphs. To support *accessibility*, the metadata and the mathematical data are stored separately. Thus it is possible to access only metadata when the original data is unavailable. Moreover, we make data accessible by providing a dump for download, and the protocol for accessing MMLKG is open, free, and universally implementable (e.g., HTTP RESTful API^21^). Thanks to structured data, thesaurus style, and the support of shared vocabularies, *interoperability* is an inherent feature of knowledge graphs. Data is defined using the core vocabulary and schema in order to get the well-structured representation of relevant information and data types. Links to other data are provided in MMLKG. Finally, we make MMLKG *reusable* by associating the data with clear provenance and by reusing popular vocabularies and ontologies (Dublin Core, SKOS, etc.). Moreover, the license of the graph data is CC-BY, which facilitates the reuse of the data.

### Interlinked and structured data best practices

Structured data stands as essential in the realm of data organization, offering a systematic framework defined by schema specifications and vocabularies. This format plays a pivotal role in the efficient provisioning of information and the methodical classification of an extensive array of content types^22^. We used the above features when building MMLKG.

Moreover, our dataset not only adheres to structured data principles but also advances its capabilities by robustly supporting internal links. This feature allows for the creation of intricate relationships within the dataset, enhancing its utility for users seeking to navigate and explore interrelated information seamlessly. Additionally, MMLKG is designed to provide the potential for linking with external data sources, thereby expanding the horizons of knowledge discovery and cross-domain insights^18^. MMLKG faithfully adheres to the foundational principles governing the publication and interlinking of structured data. It recognizes the significance of making data openly accessible and ensuring its usability for a diverse audience^23^.

Furthermore, the MMLKG dataset adopts the rigorous five-star Linked Data scheme for web-based data publishing. This scheme sets stringent data interoperability and connectivity standards, ensuring that the dataset is not only accessible but also seamlessly integrated into the broader web of knowledge^24^. We believe that MMLKG positions its dataset as a valuable resource for the research community and knowledge enthusiasts alike.

### Mizar workflow

The Mizar Mathematical Library is a repository of formal mathematical definitions and proofs used within the Mizar system. It is organized as a collection of articles verified against their logical soundness using the Mizar proof-checker. Every article comprises two parts: an import section with declarations of notions imported from the database into the current development and a section with new definitions, theorems, and other items allowed by the Mizar language^25,26^. Developed works can be verified at every stage of their creation.

The Mizar proof-checker consists of several modules that verify various aspects of the correctness of given articles. Scanner reads an input file and splits it into a sequence of tokens. Parser checks the syntactical correctness and generates a parse tree in XML-based format. MSM Processor reads the parse tree and resolves the usages of variables and labels of statements. Analyzer identifies all used mathematical concepts (the Mizar language allows overriding symbols) and generates XML-based files in which the exact information about all used notions is stored. These files, with the extension.esx, can then be taken as input files by external applications for their purposes, like cross-verification or presentation of the content of MML in different formats. When all notions are properly identified, Reasoner checks the correctness of structures of proofs (proof-skeletons), and finally, Verifier verifies the logical correctness of proofs.

**Fig. 1:**
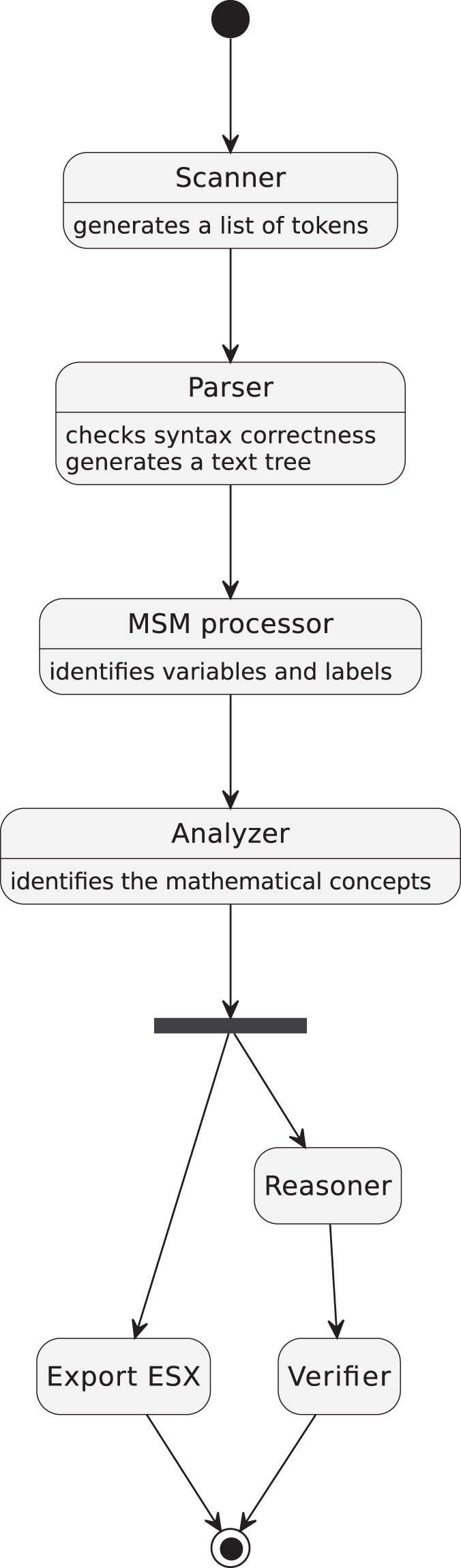
Mizar system workflow. Rounded rectangles symbolize different Mizar modules.

Mizar workflow is presented in Fig. 1.

### MMLKG Generation

To produce MMLKG, we use ESX (.esx) files generated by the Mizar verifier for the Mizar Version 8.1.11_5.68.1412 with 1415 Mizar articles. For this work, we extracted only some parts of the information accessible in the files:

- usages of all notions defined in MML within all articles stored in MML:
  - predicates, which represent relations between objects (e.g. ∈, ≤),
  - adjectives, which describe properties of objects (e.g. finite, one-to-one),
  - types, which define new types of objects (e.g. function, ring),
  - functors, which represent linguistic functions used to define operations (e.g. + , ∪ ), and
  - structures, which represent encapsulated data (e.g. addMagma),

- definitions of synonyms of predicates, synonyms of adjectives, synonyms of types, and synonyms of functors introduced in MML,
- inheritance of types introduced in the whole MML,
- inheritance of structures,
- references to local statements, external theorems, and schemes (statements with second-order free variables).

Extracted parts are encoded in CSV format.

**Fig. 2:**
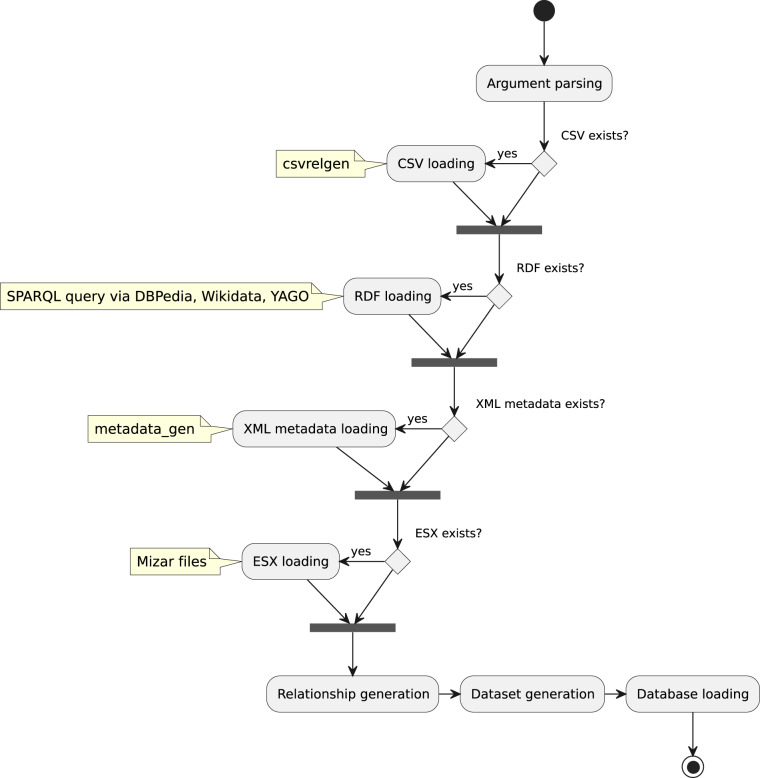
MMLKG generation stages.

Next, the Python package mizgra^27^ generates input data for MMLKG. Mizgra is a command-line tool that allows the user to choose the source data, output format, and other optional parameters. Mizgra supports external data in RDF and CSV formats. The data generation process is presented in Fig. 2 and can be conceptually divided into the following functional modules:

1. *Argument processing*: Handling user-provided arguments such as source files, preference options (including disabling or selecting specific outputs), and output format.
2. *CSV files relations loading*: If selected, loading relations data from CSV files and optionally validating the correctness of the CSV files with a series of tests.
3. *Metadata processing*: Processing automatically generated metadata (e.g., the number of definitions and the creation date) from the specified XML file or using the default file if the file has not been specified.
4. *ESX files processing*: Processing data from ESX documents based on the order specified in the mml.lar file (the file where the order of processing of the whole MML is given). Adding additional properties like “notation” and node’s “uuid”. Notations are alphanumeric codes linked to specific concepts, while the Universal Unique Identifier (UUID) identifies a node uniquely.
5. *Building relationships between ESX elements*: Finding and adding relationships among elements in single and multiple ESX documents, including MEMBER, RELATED, BROADER, and SAMEAS relationships.
  The MEMBER relationship means that an object is a part of another object. For example, “humans” are members of “mammals”. In the context of MMLKG, this is the relationship from a child to its parent ESX element.
  The RELATED relationship says that two objects are related to each other in some way without specifying the exact nature of this relationship. For example, a particular book may be related to its authors by a RELATED relationship. In MMLKG, this relationship applies to local references and references to external theorems and schemas, usages of predicates, adjectives, types (standard and structured), and operations. Most of these relationships are obtained from CSV files by default. A CSV is a text file that contains comma-separated relationship data (source file, xmlid attribute of the source file, target file, xmlid of the target file, and relationship name).
  The BROADER relationship means that an object has a broader context for another object. For example, that the object mammals has a broader concept called animals. In MMLKG, the BROADER relationship refers to the graph of structures and the tree of types and comes by default from CSV files.
  The SAMEAS relationship expresses equivalence between two objects. For example, “The United Kingdom” may have a SAMEAS relation with “UK”. This relationship is used in CSV files for synonyms of predicates, synonyms of adjectives, synonyms of types, and synonyms of operations.
6. *Building relationships to external data*: Finding and adding relationships between elements of ESX files and external data sources in RDF format. External sources can be specified by file, URL, or default data file supporting Wikidata, DBPedia, and YAGO resources. In this step, EXACTMATCH relationships are generated, which point to the same terms from external resources. Here, we also use different identifiers, such as identifiers for an object or topic in the Microsoft Academic Graph (MicrosoftAcademicID), identifiers for entries in MathWorld (MathWorldIdentifier), identifiers for a topic in Treccani’s Enciclopedia della Matematica (EnciclopediaDellaMatematicaID), etc. Moreover, we provide different links, descriptions, names, and synonyms in 40 languages.
7. *Generating output in the selected format*: Generating output data in graph formats such as GraphML.

To generate the MMLKG, mizgra version 1.0.0 was executed, specifying the ESX directory and the mml.lar file, CSVs relations directory with CSV relations validation. The ESX files and the mml.lar file are available in the repository^28^, external CSV relations files are available in the Figshare^29^, generated with the csvrelgen^30^. The metadata file is generated with metadata_gen^31^.

The command used to generate the metadata file:


mvn clean package && java -jar target/metadata_gen-1.0-SNAPSHOT-jar-with-dependencies.jar /path/to/esx_mml


The command used to generate CSV relations files:

./csvrelgen.sh

The command used to generate the dataset:


mizgra /path/to/esx_mml /path/to/mml.lar -m /path/to/metadata.xml -c /path/to/CSVs -cv > mmlkg.graphml


## Data Records

The dataset is published on Figshare^29^. The dataset file is written in GraphML^32^ and includes supporting files:

- README.md – a dataset description file,
- LICENSE.txt – a license for all files,
- stats.json – a JSON file with metadata and stats for mmlkg.graphml,
- mmlkg.graphml – a main file – GraphML of Mizar Mathematical Library Knowledge Graph for the load to different graph databases, e.g., Neo4j,./csvs/* – CSV files with relationships for the graph,./metadata/metadata.xml – an XML file for graph metadata,./metadata/metadata.xsd – a main schema for metadata.xml,./metadata/dc.xsd – Dublin Core (metadata standard for digital resources) schema for metadata.xml,./rdf/rdf_data.nt – an N-Triples (RDF serialization) file that stores standalone data grabbed from external RDF resources.

The files are generated with the following tools:

- mizgra^27^ 1.0.0 – a main tool for generating a property graph serialization,
- metadata_gen^31^ 1.0.0 – a tool for generating a property graph metadata,
- csvrelgen^30^ 1.0.0 – a tool for creating complex relationships between concepts.

The dataset is available via Cypher endpoint at https://mmlkg.uwb.edu.pl/.

## Technical Validation

### Syntactical correctness and validation

In the first step, we check the syntax of ESX documents. ESX, as an XML-based format, must adhere to the syntax rules specified by the XML specification. All ESX files are well-formed documents. XML document formatting and basic syntax rules are described and enforced through a second step called XML validation. In our case, a valid document respects the rules dictated by a particular XML schema. All ESX documents are valid. The schema document (mizar_esx.xsd) is published on GitHub^28^.

The same procedure applies to other XML files, such as metadata, used to build the knowledge graph. The XML Schema files for this document are published on Figshare^29^.

Our data is devised to be reproducible and accessible. It is possible to test its syntactical correctness using xmllint (https://gnome.pages.gitlab.gnome.org/libxml2/xmllint.html):


for i in esx_mml/*.esx; do xmllint --noout --schema./mizar_esx.xsd $i; done


### Semantic correctness and information preservation

Semantic correctness indicates that the output of a dataset mapping is always a valid dataset. In this sense, we can say that it is the correctness of data mapping.

Before we started translating ESX files, we checked the semantic correctness of these files by generating Mizar scripts in the Mizar language based on ESX files and full verification of the reproduced files. All these new files are correct.

Information preservation indicates that for some data mapping DM, there exists an “inverse” data mapping DM^−1^, which allows recovering data transformed with DM. Information preservation is fundamental because it guarantees that a database mapping does not lose information^33,34^. Moreover, it implies that the information capacity of the target dataset model subsumes the information capacity of the source dataset model.

Our dataset meets both of the above characteristics. It has been checked with a Java application available on Zenodo^35^.

One can test semantics and information preservation using our cross-platform tool:


mvn clean package &&./esx_verify.sh download mml.lar


## Usage Notes

### Dataset loading

Our dataset is delivered in the well-known GraphML format^32^. MMLKG can be imported into various databases, e.g. Neo4j^36,37^, Oracle Database^38^, Amazon Neptune^39^, JanusGraph^40^, OrientDB^41^, etc. We used Neo4j 5.9.0+ to demonstrate and provide the endpoint https://browser.mmlkg.uwb.edu.pl/browser/; authentication type: no authentication.

### Competency questions

Based on the domain and scope of the Mizar Mathematical Library, we determine the specific information needs that users may have about our dataset. In the next step, based on the identified information needs, we formulate a set of Competency Questions^42^. Then, we write Cypher queries^43^ that correspond to each competency question. These queries can be executed in Neo4j. The Competency Questions and Cypher queries are presented below.

1. What is a direct super-type of the type Element?
  MATCH (n:‘Mode-Pattern‘)-[:BROADER]- > (o:‘Mode-Pattern‘)WHERE n.spelling = ‘Element’RETURN n, o, n.absolutepatternMMLId, o.spelling, o.absolutepatternMMLId
  An example of this query result is shown in Fig. 3.

**Fig. 3:**
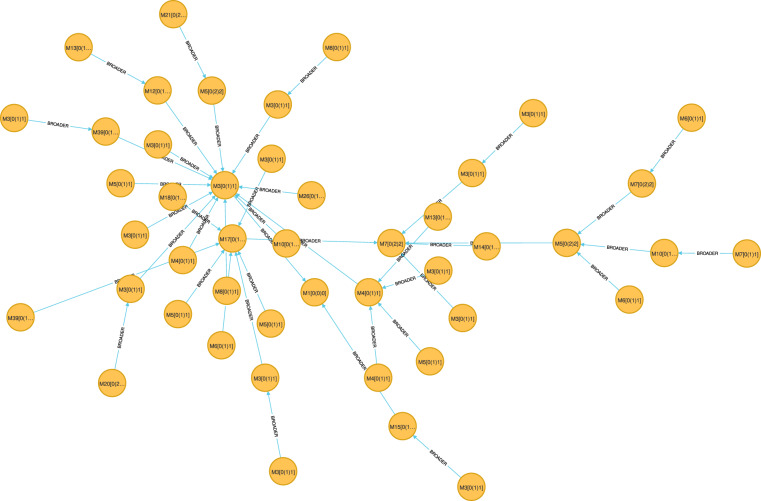
Fragment of the query 1.
2. What are the direct super-structures of the structure ZeroStr?
  MATCH (n:‘Structure-Pattern‘)-[:BROADER]- > (o:‘Structure-Pattern‘)WHERE n.spelling = ‘ZeroStr’RETURN n.absolutepatternMMLId, o.spelling, o.absolutepatternMMLId
3. Which adjectives are mostly used as negated ones?
  MATCH (n:Attribute)WHERE n.nonocc IS NOT NULLWITH n.absolutepatternMMLId AS attribute, n.spelling AS spelling,COUNT(n) AS occurrencesRETURN attribute, spelling, occurrences
4. Which predicates are redefined mostly?
  MATCH (p1:‘Predicate-Pattern‘)-[:MEMBER]- > (d1:‘Predicate-Definition‘)< -[:MEMBER]-(r1:Redefine),(p2:‘Predicate-Pattern‘)-[:MEMBER]- > (d2:‘Predicate-Definition‘)< -[:MEMBER]-(r2:Redefine)WHERE r1.occurs = “false” AND r2.occurs = “true” ANDp1.absolutepatternMMLId = p2.absoluteorigpatternMMLIdRETURN p1.spelling as symbol, p1.absolutepatternMMLId as definition,COUNT(*) as occurrencesORDER BY occurrences DESC
5. Which redefinitions of the equality ( = ) exist?
  MATCH (p:‘Predicate-Pattern‘)-[:MEMBER]- > (d:‘Predicate-Definition‘)< -[:MEMBER]-(r:Redefine)WHERE r.occurs = “true” AND p.spelling = “ = “RETURN p.absolutepatternMMLId AS redefinition
6. What theorems involve the type Nat?
  MATCH (t:‘Theorem-Item‘)< -[:MEMBER]-(p:Proposition) < -[:MEMBER*]-(s:‘Standard-Type‘)WHERE s.spelling = “Nat”RETURN t.MMLId AS theoremID
7. What theorems exist about the type Nat and the relation <  = ?
  MATCH (t:‘Theorem-Item‘) < -[:MEMBER]-(p:Proposition)< -[:MEMBER*]-(s:‘Standard-Type‘)WHERE s.spelling = “Nat”WITH collect(t) AS set1MATCH (t:‘Theorem-Item‘) < -[:MEMBER]-(p:Proposition)< -[:MEMBER*]-(r:‘Relation-Formula‘)WHERE r.spelling = “ <  = “WITH set1, collect(t) AS set2WITH [x IN set1 WHERE x IN set2] AS intersectionUNWIND intersection AS interRETURN DISTINCT inter.MMLId AS theoremID
8. Which synonyms exist for types?
  MATCH (m:‘Mode-Synonym‘) < -[:MEMBER]-(n:‘Mode-Pattern‘),(m:‘Mode-Synonym‘) < -[:MEMBER]-(:‘Pattern-Shaped-Expression‘)< -[:MEMBER]-(x:‘Mode-Pattern‘)RETURN n.spelling AS synonym, x.spelling AS original
9. What are all the usages of the theorem labeled JORDAN:21?
  MATCH (r:‘Theorem-Reference‘)-[:MEMBER*]- > (t:‘Theorem-Item‘)WHERE r.MMLId = “JORDAN:21”RETURN t.MMLId AS theoremID
10. What are all types of the functor labeled SUBSET_1:1?
  MATCH (m:‘InfixFunctor-Pattern‘)-[:MEMBER]- > (o)< -[:MEMBER]-(s:‘Type-Specification‘) < -[:MEMBER]-(t:‘Standard-Type‘)-[:RELATED]- > (p:‘Mode-Pattern‘)-[:BROADER*]- > (z)WHERE m.absolutepatternMMLId = ‘SUBSET_1:1’RETURN distinct t.absolutepatternMMLId AS typeID, t.spelling AS type,z.spelling AS superType, m, o, s, t, p, z
  An example of this query result is shown in Fig. 4.

**Fig. 4:**
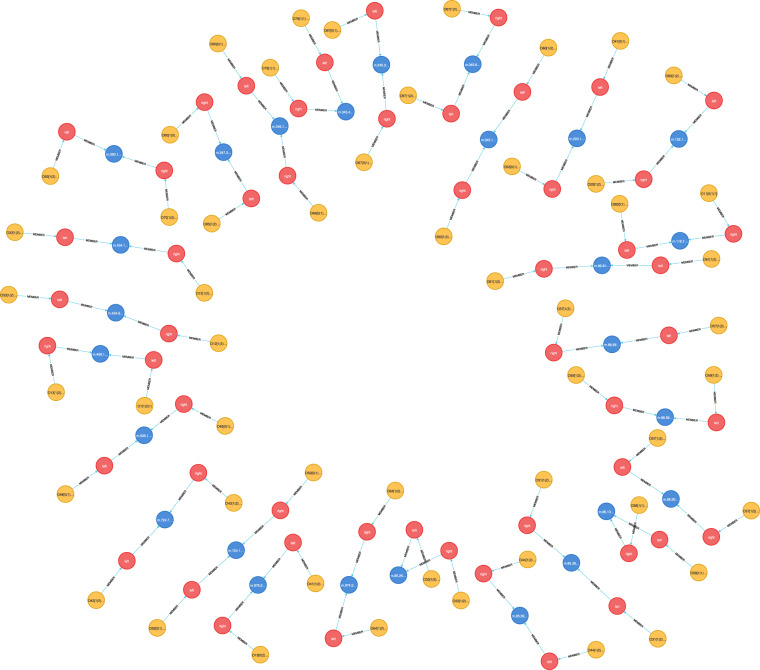
Fragment of the query 10.
11. What adjectives are assigned to the operation [:?
  MATCH (x) < -[:MEMBER]-(n:‘Circumfix-Term‘),(x) < -[:MEMBER]-(c:‘Adjective-Cluster‘) < -[:MEMBER]-(a)WHERE n.spelling = ‘[:’RETURN n.id AS nodeID, a.nonocc AS negated, a.spellinga.absolutepatternMMLId AS absolutepatternMMLId
12. What adjectives are assigned to the adjective empty when applied to the type set?
  MATCH (a:‘Text-Proper‘) < -[:MEMBER*]-(r:‘Conditional-Registration‘)< -[:MEMBER]-(c1:‘Adjective-Cluster‘) < -[:MEMBER]-(a1:Attribute),(r:‘Conditional-Registration‘) < -[:MEMBER]-(c2:‘Adjective-Cluster‘)< -[:MEMBER]-(a2:Attribute),(r:‘Conditional-Registration‘) < -[:MEMBER]-(t:‘Standard-Type‘)WHERE c1.role = “antecedent” AND c2.role = “consequent”AND a1.spelling = “empty” AND a1.nonocc IS NULL AND t.spelling = “set”RETURN a2.spelling AS spelling, a.articleid AS articleIDORDER BY articleIDLIMIT 15
13. What articles are associated with term reductions of the operation *?
  MATCH (a:‘Text-Proper‘) < -[:MEMBER*]-(:Redex) < -[:MEMBER]-(i:‘Infix-Term‘)WHERE i.spelling = “*“RETURN a.articleid AS articleID ORDER BY articleIDORDER BY articleID
14. What are all the term identification rules? – An example of this query result is shown in Fig. 5.

**Fig. 5:**
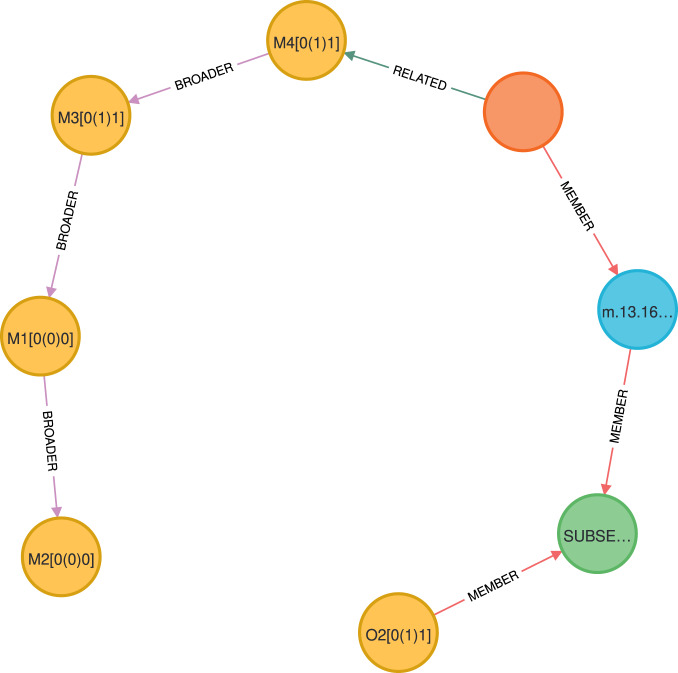
Fragment of the query 14.
  MATCH (a:‘Text-Proper‘) < -[:MEMBER*]-(i:Identify)< -[:MEMBER]-(p:‘Pattern-Shaped-Expression‘)< -[:MEMBER]-(f:‘InfixFunctor-Pattern‘),(i:Identify) < -[:MEMBER]-(r:‘Pattern-Shaped-Expression‘)< -[:MEMBER]-(g:‘InfixFunctor-Pattern‘)WHERE p.role = “left” AND r.role = “right”RETURN a.articleid AS articleID, f.spelling AS spelling,g.spelling AS orgSpelling, i, p, f, r, g
15. What is the total number of articles in the dataset?
  MATCH (n:TextProperCard) RETURN n.value
16. Who are the creators of the dataset?
  MATCH (n)-[:HAS]- > (m:creator)RETURN m, n, m.value, n.id
17. What is a description in Polish of the Jordan Curve Theorem that can be found in external sources?
  MATCH (n:‘Notion-Name‘)-[d:description]- > (m)WHERE n.inscription = “Jordan Curve Theorem” AND d.lang = “pl”RETURN m.value
18. Which external sources provide information about the Fundamental Theorem of Algebra?

MATCH (n:‘Notion-Name‘)-[:exactMatch]- > (m)WHERE toLower(n.inscription) = “fundamental theorem of algebra”RETURN m.value


### Potential applications

In this Subsection, we explore a range of practical and innovative ways in which the concepts and technologies discussed can be put into use across various domains. Potential applications are:

- Semantic search: Leveraging a knowledge graph enhances semantic search in the field of mathematics, aiming to deliver highly accurate and context-aware online search results, thereby improving the comprehension of user intent when users seek to discover relevant scientific materials or expand their queries with related concepts and synonyms, facilitating the formalization and writing new mathematical works. In particular:
  - Resource discovery: Semantic search allows learners to quickly discover relevant educational materials. When students search for a specific mathematical topic, semantic search algorithms consider not only keyword matches but also semantic relationships between concepts.
  - Query expansion: Semantic search can expand the user’s query by including related concepts and synonyms, improving chances of finding relevant results, what is desirable in the process of formalizing and writing new works.
- Education and e-learning: In the realm of education and e-learning, enhancing the learning experience can be achieved by leveraging semantic search and knowledge graphs, which improve access to mathematical educational resources through better organization and the creation of more intuitive interfaces, allowing learners to quickly discover relevant materials and cross-reference related mathematical content. In particular:
  - Organizing educational materials: Knowledge graphs can be used to organize vast amounts of educational content, including textbooks and articles.
  - Cross-referencing resources: Knowledge graphs allow for cross-referencing related mathematical materials.
- Databases and data warehouses: In the realm of database and data warehouse management, knowledge graphs find significant applications, particularly when it comes to dealing with large mathematical datasets. They play a pivotal role in facilitating the management of extensive mathematical data collections and enhancing data quality by structuring and organizing mathematical knowledge effectively through data integration from various sources and data enrichment using external knowledge sources like Mizar and Wikipedia. In particular:
  - Data integration: Knowledge graphs can integrate data from various sources, including structured databases, unstructured text, and external APIs. Linking data points through semantic relationships creates a unified view of the information landscape. In this case, our tool combines information from various sources, e.g., Mizar, Wikipedia, etc.
  - Data enrichment: Knowledge graphs can enrich existing mathematical data by linking it to external knowledge sources. This enrichment adds context and additional attributes to the data, enhancing its quality and usefulness.
- Scientific research: Contextualized information retrieval in the field of mathematics is greatly enhanced by semantic search, as it understands the context of research queries, making it easier for researchers to find precise mathematical information and present them, while also utilizing knowledge graphs to create concept maps that visually represent the relationships between scientific mathematical concepts, theories, and ideas, enriching existing mathematical data with context and additional attributes to enhance its quality and usefulness in research endeavors.
  - Concept mapping: Knowledge graphs can be used to create concept maps that visually represent the relationships between scientific concepts, theories, and ideas. These maps help researchers gain a deeper understanding of the field and identify research gaps.
  - Contextualized information retrieval: Semantic search makes it easier for researchers to find precise mathematical information and present them based on understanding the context of research queries.

## Data Availability

The code to reproduce results, documentation, and tutorials are available in the GitHub repositories: metadata_gen^31^ 1.0.0 – a tool for generating a property graph metadata, csvrelgen^30^ 1.0.0 – a tool for creating complex relationships between concepts, mizgra^27^ 1.0.0 – a main tool for generating a property graph serialization, esx_verify^35^ 1.0.1 – a tool for checking semantics correctness. All software is provided with open-source licenses and has a Docker version. A sample usage contains: 1. In the first step, users should use ESX files. 2. In the second step, users can optionally check syntactical correctness via xmllint or other XML Schema validators. 3. In the third step, users can optionally check semantic correctness via esx_verify. 4. In the fourth step, users may use csvrelgen to generate relationships. 5. In the fifth step, users can use metadata_gen to generate metadata (in XML) for the dataset. 6. In the sixth step, users may prepare RDF resources, e.g. via the SPARQL endpoint. 7. In the seventh step, users can use ESX (see item 1), CSV (see item 4), XML metadata (see item 5), and RDF (see item 6). 8. In the last step, users can load the GraphML data to the graph database, e.g. Neo4j^37^.
